# *FAS* rs2234767 and rs1800682 polymorphisms jointly contributed to risk of colorectal cancer by affecting SP1/STAT1 complex recruitment to chromatin

**DOI:** 10.1038/srep19229

**Published:** 2016-01-13

**Authors:** Shizhi Wang, Shenshen Wu, Qingtao Meng, Xiaobo Li, Jinchun Zhang, Rui Chen, Meilin Wang

**Affiliations:** 1 Key Laboratory of Environmental Medicine Engineering, Ministry of Education, School of Public Health, Southeast University, Nanjing, China; 2 Department of Medical Laboratory, Nanjing Municipal Hospital of TCM, Nanjing, China; 3 Department of Environmental Genomics, Jiangsu Key Laboratory of Cancer Biomarkers, Prevention and Treatment, Cancer Center, Nanjing Medical University, Nanjing, China; 4 Department of Genetic Toxicology, the Key Laboratory of Modern Toxicology of Ministry of Education, School of Public Health, Nanjing Medical University, Nanjing, China

## Abstract

*FAS* rs2234767 (−1377 G>A), rs1800682 (−670 A>G) and *FASLG* rs763110 (−844 C>T) promoter polymorphisms can influence transcriptional activities of the genes and thus multiple tumors susceptibility. To investigate their association with risk of colorectal cancer (CRC), the three SNPs were genotyped in 878 cases and 884 controls and the results showed that the *FAS* rs2234767 and rs1800682 were in a high linkage disequilibrium (LD) with each other (*D*’ = 0.994) and jointly contributed to an increased risk of CRC (without vs. with rs2234767 GG/rs1800682 AA genotypes, adjusted OR = 1.30, 95% CI = 1.05 − 1.61). *In vivo* ChIP assays evaluated the effect of rs2234767 and rs1800682 on recruitment of SP1 and STAT1, respectively, to chromatin. The results showed SP1 interacting specifically with STAT1 recruited to their respective motifs for transcriptional activation. The mutant alleles rs2234767 A and rs1800682 G jointly affected coupled SP1 and STAT1 recruitment to chromatin. The interplay between SP1 and STAT1 was critical for the functional outcome of rs2234767 and rs1800682 in view of their high LD. In conclusion, the *FAS* rs2234767 and rs1800682 polymorphisms were in high LD with each other, and they jointly contributed to an increased risk of CRC by altering recruitment of SP1/STAT1 complex to the *FAS* promoter for transcriptional activation.

Colorectal cancer (CRC) is the third most commonly diagnosed cancers worldwide, accounting for roughly 1.2 million new cases and 600,000 deaths per year^1^. CRC is a complex disease resulting from both genetic and epigenetic alterations, including genetic variants^2^ and abnormal DNA methylation patterns^3^,^4^,^5^, among others. Two decades of research on genetic architecture of CRC has revealed that inherited susceptibility is a major component of CRC predisposition, with genetic factors accounting for 12–35% risk of CRC^2^.

A large number of studies have implicated the involvement of deregulated apoptosis pathway in CRC carcinogenesis^6^,^7^. Defect, dysfunction or altered expression of genes encoding key apoptotic proteins modify risk of CRC^8^. FAS, also known as CD95, encoded by *FAS* gene, is a cell surface factor and important inducer of the extrinsic apoptosis signaling pathway. FAS ligand, FASLG, also known as CD95L, encoded by *FASLG* gene, is a member of the tumor necrosis factor superfamily. FASLG binding to FAS triggers apoptosis through activation of CASP8^9^. Accumulating evidence suggests that altered expression of FAS and/or FASLG contributes to development of CRC^8^.

Functional SNPs within the promoter region of gene are capable of affecting transcription and subsequently modulating risk of disease^10^,^11^. It has been reported that there are two functional SNPs in the promoter of *FAS* gene (*FAS* −1377 G>A, rs2234767; −670 A>G, rs1800682), which located within the consensus sequences of the SP1 and STAT1 transcription factors (TF) binding sites, respectively^12^. Sibley *et al.*^13^ reported that the rs2234767 A had a greatly reduced ability to bind SP1 compared with rs2234767 G and people with A allele had a significantly increased risk of acute myeloid leukemia (AML); however, both the rs1800682 A and rs1800682 G alleles could bind STAT1 and have no detectable difference in binding affinity.

Wu *et al.*^14^ first identified a T to C substitution at position −844 in the promoter of *FASLG* gene (*FASLG* −844 C>T, rs763110), which located in a putative binding motif for CAAT/enhancer-binding protein β (C/EBPβ). Functional study revealed that −844 C allele could increase basal FASLG expression, suggesting the −844 C>T polymorphism may affect the FASLG-mediated apoptotic signaling. A number of studies have been conducted to investigate the association between the three SNPs and a variety of tumors, including esophageal squamous-cell carcinoma (ESCC)^15^,^16^, squamous cell carcinoma of the head and neck (HNSCC)^17^, bladder cancer^18^, and gastric cancer^19^.

In this study, we aimed to determine the association of the *FAS* rs2234767, rs1800682, and *FASLG* rs763110 polymorphisms with risk of CRC in a Chinese population and the molecular mechanism underlying the association.

## Methods and Materials

### Ethics statement

The study was approved by the institutional review board of Southeast University. Each subject signed an informed consent. The research protocol was carried out in accordance with the approved guidelines.

### Patients and samples

A total of 878 CRC patients and 884 healthy controls were enrolled in this study. The detailed information on the subjects has been described elsewhere^20^. Briefly, all patients were recruited from the First Affiliated Hospital of Nanjing Medical University between September 2010 and October 2013. The pathological stage of CRC at the time of diagnosis was classified into Dukes A, B, C and D. All controls were genetically unrelated to the cases and recruited from those who were seeking for health care in the same hospital. After signed the informed consent, all subjects donated 5 ml of venous blood sample for genomic DNA extraction.

### Genotyping

The genotyping of *FAS* rs2234767, rs1800682 and *FASLG* rs763110 was performed by TaqMan allelic discrimination method equipped with ABI 7900 HT Real Time PCR System (Applied Biosystems, CA, USA). The reaction conditions were set as follows: 95 °C for 10 min followed by 40 cycles of 95 °C for 15 sec, and 60 °C for 1 min. At least 10% of the samples were randomly selected for genotyping confirmation, and the results were 100% concordant.

### Chromatin immunoprecipitation assay (ChIP)

Human peripheral white blood cells (5 × 10^7^ per sample) were fixed for 10 min at 37 °C with 4% formaldehyde. After incubation, fresh glycine was added to a final concentration of 125 mM to stop cross-linking. After 5 min at room temperature, the samples were pelleted in an centrifuge at 3600 rpm (2000 g) for 2 min at 4 °C, washed once with cold PBS plus protease inhibitors, and then repelleted. The pellet was resuspended in 1 ml of PBS and ground the cells using a micro-tissue grinder on ice. Cells were pelleted again as above at 4 °C. ChIP was performed using the ChIP-IT^TM^ Express Magnetic assay kit (Cat. No. 53009, Active Motif). The antisera for ChIP reaction was normal mouse IgG (Cat. No. 2027, Santa Cruz Biotechnology, Inc.), normal rabbit IgG (Cat. No. NI01, EMD Chemicals, Inc., Gibbstown, NJ), anti-human SP1 (Cat. No. 9389, Cell Signaling Technology), and anti-human STAT1 (Cat. No. 9172, Cell Signaling Technology). Precipitated genomic DNA was analyzed by quantitative PCR in triplicate measurements for each sample using the following human *FAS* promoter primers: 5′-ACCATCCTCCTTATCCCACT-3′ (forward) and 5′-GTAGGTGTTGATAGGCTTGA-3′ (reverse) for rs2234767; 5′-CTAAGGGGCCCTCCCTTTT-3′ (forward) and 5′-ACTTGCGGGGCATTTGACT-3′ (reverse) for rs1800682. Captured genomic DNA was normalized to input material and the samples with different genotypes compared.

### Statistical analysis

The Hardy-Weinberg equilibrium (HWE) of the controls’ genotype frequencies was evaluated by a goodness-of-fit chi-square test (χ^2^ test). Bonferroni correction for multiple testing was also applied. Crude and adjusted odds ratios (ORs) and 95% confidence intervals (CIs) were calculated to analyze the magnitude of the association between the genotypes and risk of CRC by univariate and multivariate unconditional logistic regression models, respectively. A *P*-value < 0.05 was considered statistically significant.

## Results

### Association between the FAS and FASLG polymorphisms and risk of CRC

The genotype frequencies of *FAS* rs2234767, rs1800682 and *FASLG* rs763110 among the controls were all in agreement with Hardy-Weinberg equilibrium (*P* = 0.11 for rs2234767, 0.060 for rs1800682 and 0.53 for rs763110). As shown in Table 1, the frequencies of rs2234767 mutant A allele was higher in the cases than the controls (39% and 34%, *P* = 0.013 after Bonferroni correction). Moreover, the frequencies distribution of rs2234767 genotypes were significantly different between the cases and controls (*P* = 0.0051), which remained significant after Bonferroni correction (*P* = 0.015). When the rs2234767 GG genotype used as the reference, the heterozygous GA and AA genotypes were both associated with significantly increased risk of CRC (adjusted OR = 1.39, 95% CI = 1.13 − 1.71 for the GA genotype; 1.37, 1.02 − 1.84 for the AA genotype), and the risk did not change substantially under the assumption of a dominant genetic model (adjusted OR = 1.38, 95% CI = 1.14 − 1.68).

The genotype and allele frequencies distribution of the *FASLG* rs763110 were not significantly different between the cases and controls (*P*_genotype_ = 0.71 and *P*_allele_ = 0.92; *P*_genotype_ = 1.0 and *P*_allele_ = 1.0 after Bonferroni correction). The allele frequency distribution of the *FAS* rs1800682 was not significantly different between the cases and controls (*P*_allele_ = 0.13 and *P*_allele_ = 0.39 after Bonferroni correction). The difference of the rs1800682 genotype distribution was Quasi significant between the cases and controls (*P* = 0.060); however, the difference did not remain significant after Bonferroni correction (*P* = 0.18). Further analysis showed that the rs1800682 polymorphism was associated with an increased risk of CRC under the dominant genetic model (AG/GG vs. AA, adjusted OR = 1.26, 95% CI = 1.04 − 1.53).

### Association between the combined genotypes of the FAS polymorphisms and risk of CRC

Linkage disequilibrium analysis (LD) revealed a strong LD between the two *FAS* polymorphisms among the controls (*D*’ = 0.994 and *r*^2^ = 0.848, *P* < 0.001), suggesting a joint effect between the two *FAS* polymorphisms. To evaluate the genotype- genotype interaction, we dichotomized the FAS genotypes as either rs2234767 GG or rs2234767 GA/AA and rs1800682 AA or rs1800682 AG/GG. When the rs2234767 GG/rs1800682 AA genotypes used as the reference, the (rs2234767 GA/AA)/(rs1800682 AA) genotypes and the (rs2234767 GA/AA)/(rs1800682 AG/GG) genotypes were associated with a significantly higher risk of CRC (adjusted OR = 15.49, 95% CI = 2.01 − 119.25 for the (rs2234767 GA/AA)/(rs1800682 AA) genotypes; and 1.30, 1.06 − 1.59 for the (rs2234767 GA/AA)/(rs1800682 AG/GG) genotypes; Table 2).

### Stratification analysis of the association of FAS combined genotypes with CRC susceptibility by demographic variables

To control the impact of confounders on the genetic association, we performed stratification analysis. The combined genotypes were dichotomized into two groups, i.e., with rs2234767 GG/rs1800682 AA and without rs2234767 GG/rs1800682 AA, to facilitate further analysis. As shown in Table 3, compared with the rs2234767 GG/rs1800682 AA genotypes, the individuals carrying the combined genotypes without rs2234767 GG/rs1800682 AA had a higher risk of CRC (adjusted OR = 1.28, 95% CI = 1.05 − 1.56), and the risk was more pronounced among the subgroups of age >60 years, female, never smokers or drinkers, having no family history of cancer (adjusted OR = 1.50, 95% CI = 1.13 − 1.99 for >60 years, 1.77; 1.26 − 2.47 for female; 1.39, 1.09 − 1.77 for never smokes; 1.33, 1.05 − 1.67 for never drinkers; and 1.34, 1.08 − 1.67 for people having no family history of cancer).

### Association between the combined genotypes of the FAS polymorphisms and progression of CRC

We further evaluated the association between the *FAS* combined genotypes and grade and stage of CRC. When compared with the rs2234767 GG/rs1800682 AA genotypes, the combined genotypes without rs2234767 GG/rs1800682 AA were associated with a significantly increased risk of CRC with intermediate grade (adjusted OR = 1.30, 95% CI = 1.05 − 1.61; Table 4). However, the combined genotypes were not significantly associated with CRC with low or high grade, which was likely due to the reduced number of subjects. In the stratification of stage, a significantly increased risk was only found between the combined genotypes without rs2234767 GG/rs1800682 AA and CRC with Dukes C and D stage (adjusted OR = 1.33, 95% CI = 1.04 − 1.71; Table 4).

### rs2234767 A coordinates with rs1800682 G to attenuate binding affinity of SP1 and STAT1 to FAS promoter *in vivo*

To determine the molecular mechanisms underlying the association of *FAS* polymorphisms with an increased risk of CRC, the effect of the rs2234767 and rs1800682 polymorphisms on the ability of SP1 and STAT1 transcription factors to bind the endogenous *FAS* promoter region was assessed by ChIP assays. Because the rs2234767 G and rs1800682 A alleles were in high LD with each other as mentioned above, there existed only three combined genotypes in our samples for ChIP assays. Finally, a total of 9 people with three different combined genotypes, i.e., rs2234767 GG/rs1800682 AA, rs2234767 GA/rs1800682 AG, and rs2234767 AA/rs1800682 GG, were selected for ChIP assays.

**Figure 1:**
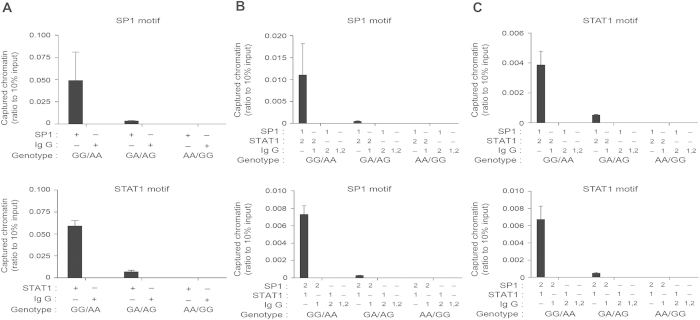
*FAS* rs2234767 A and rs1800682 G alleles affect coupled SP1 and STAT1 recruitment to chromatin. (**A**) Chromatin immunoprecipitation (ChIP) of the *FAS* promoter with three different genotypes (rs2234767 GG/rs1800682 AA, rs2234767 GA/rs1800682 AG and rs2234767 AA/rs1800682 GG) using antibody for SP1 and STAT1(single pool generated from triplicate biological samples/manipulation; triplicate measurements/pool; mean ± SE). (**B**) Sequential ChIP of the *FAS* promoter containing SP1 motif immunoprecipitated first using antibody for SP1 followed by antibody for STAT1 (upper) or first using antibody for STAT1 followed by antibody for SP1 (lower). (**C**) Sequential ChIP of the *FAS* promoter containing STAT1 motif immunoprecipitated first using antibody for SP1 followed by antibody for STAT1 (upper) or first using antibody for STAT1 followed by antibody for SP1 (lower). For all combined genotypes in the figure, left genotype arises from the rs2234767 and right from the rs1800682 polymorphism.

As shown in Fig. 1A, the region with rs2234767 G and rs1800682 A alleles were readily immunoprecipitated by anti-SP1 and anti-STAT1, respectively, but not IgG, demonstrating SP1 and STAT1 interact specifically with the *FAS* promoter in chromatin (left). Interestingly, the ability of SP1 and STAT1 to bind the *FAS* promoter was dramatically decreased with the increase of mutant alleles. The amount of chromatin with the rs2234767 GA/rs1800682 AG genotypes captured by anti-SP1 and anti-STAT1 was 6.9% and 12%, respectively, of that with the rs2234767 GG/rs1800682 AA genotypes (middle). The homozygous mutant genotypes rs2234767 AA/rs1800682 GG eliminated both the SP1 and STAT1 recruitment to the *FAS* promoter (right). Furthermore, as assessed by sequential ChIP (Re-ChIP), the SP1 interacting with STAT1 was recruited to the SP1 (Fig. 1B, left) and STAT1 motifs (Fig. 1C, left) for transcriptional regulation. The mutant alleles could also eliminate the SP1/STAT1 complex recruitment to the SP1 (Fig. 1B, middle and right) and STAT1 motifs (Fig. 1C, middle and right). We also selected 9 other people with three combined genotypes for reproducibility of ChIP assays, and found the results were consistent with those mentioned above (Fig. S1). Our results indicated that SP1 and STAT1 contributed equally to activate the transcription of FAS in CRC and the interplay between these factor was critical for the functional outcome of *FAS* rs2234767 and rs1800682 in view of their high LD.

## Discussion

In the present study, we analyzed the association of *FAS* rs2234767, rs1800682 and *FASLG* rs763110 polymorphisms with risk of CRC in a Chinese population. The *FAS* rs2234767 and rs1800682 polymorphisms had effect on increasing risk of CRC and a joint effect on risk and progression, but not for the *FASLG* rs763110 polymorphism. The joint effects of the two FAS polymorphisms on risk of CRC were more pronounced among the subgroups with age >60 years, female, never smokers, never drinkers, having no family history of cancer, and CRC with intermediate grade and with Dukes C and D stage. Functional studies revealed that the SP1 interacting with STAT1 was recruited to the SP1 and STAT1 motifs within the promoter of *FAS* for transcriptional regulation, and the interplay between these factor was critical for the functional outcome of *FAS* rs2234767 and rs1800682 in view of their high LD. Given the role of FAS/FASLG pathway in carcinogenesis, it is biologically plausible that the rs2234767 and rs1800682 polymorphisms may modulate the risk of CRC by attenuating SP1/STAT1 complex-mediated transcriptional activation of *FAS*, which in turn dampening FAS apoptotic pathway.

Colorectal cancer is a complex disease and develops through a multistage process^21^,^22^. During the process, colorectal epithelial cells accumulate a number of molecular changes and eventually become fully malignant cells. These molecular changes involve mutations in the well-defined genes or pathways such as *APC*, mismatch repair genes like *MLH1*, and *SMAD4*, and epigenetic changes such as global DNA hypomethylation in repetitive sequences (satellite and LINE repeats) and promoter hypermethylation of tumor suppressor genes like MLH1, RUNX3 and SEPT9^3^,^4^,^5^. It is now well accepted that genes that regulate apoptosis are important variables in cancer development. A lot of studies have shown that alteration of FAS and FASLG expression decreases the apoptotic activity and facilitates tumor cells evading or suppressing the immune system^23^,^24^. Deregulated FAS and FASLG expression are common features of most human malignancies and associated with progression of a variety of tumors, including CRC^24^,^25^,^26^. Therefore, the functional variants of the *FAS* and *FASLG* genes which were capable of influencing their expression could be expected to have effect on cell death and thus, carcinogenesis.

*FAS* rs2234767 and rs1800682 polymorphisms have been reported to be able to alter the SP1 and STAT1 binding site, respectively, leading to dysregulated FAS expression^13^,^14^. A lot of studies have investigated the relationship between these functional SNPs and risk of diseases including tumors such as esophageal cancer^16^, HNSCC^17^, leukemia^27^, and gastric cancer^28^. However, the studies on the association between these SNPs and risk of CRC are limited. Our data showed that the functional *FAS* rs2234767 G>A and rs1800682 A>G polymorphisms were associated with significantly increased risk of CRC. Yang *et al.*^29^ also reported that the *FAS* rs2234767 polymorphism was associated with an risk of CRC, which was consistent with our results. In a meta-analysis of 52 studies, Xu *et al.*^30^ found that the carriers of the *FAS* rs2234767 A are more susceptible to the majority of cancers than non-carriers, which further corroborated our conclusions.

Although the *FAS* polymorphisms have been widely investigated their association with multiple tumors susceptibility^30^,^31^,^32^, the studies on mechanisms underlying the association were limited. The rs2234767 and rs1800682 polymorphisms can alter the SP1 and STAT1 binding site, leading to downregulated FAS expression^13^,^14^. It is reported that occupation of contiguous DNA-binding sites for STAT1 and SP1 are both required for full activation of the ICAM-1 by IFN-γ^33^. Similarly, we also found that the SP1 interacting with STAT1 was recruited to the SP1 and STAT1 motifs within the promoter of *FAS* for transcriptional regulation by ChIP assays. Moreover, the mutant alleles rs2234767 A and rs1800682 G could eliminate the SP1/STAT1 complex recruitment to the SP1 and STAT1 motifs. Our ChIP assays indicated that the interplay between the two transcription factors was critical for the functional outcome of *FAS* rs2234767 and rs1800682 in view of their high LD.

Recently, a relatively new field of epidemiology named molecular pathological epidemiology (MPE) has emerged as an integrative interdisciplinary field of molecular pathology and epidemiology, the concept of which has been consolidated by Ogino and Stampfer^34^,^35^. In MPE, a particular exposure including genetic factor is evaluated in relation to a specific somatic molecular change to better understanding its role in the carcinogenic and pathologic process. Moreover, an interactive effect of tumorous molecular features and the exposures of interest on tumor behavior can gain insights into tumor molecular changes, which may be predictive or prognostic tissue biomarkers^35^. Therefore, MPE can shed light on the pathogenic process and help optimize personalized prevention and therapy. MPE requires multidisciplinary collaboration between epidemiology, pathology, bioinformatics, biostatistics, and computational biology. Hence, the Second International MPE Meeting was held in Boston in December 2014 to discuss measures to address challenges and move this field forward, especially initiating the effort of specifying guidelines for MPE (“STROBE-MPE”) as first proposed in 2012^36^,^37^. Making consensus guidelines facilitating study reporting contributes to building a field and enhancing its contributions, such as the development of the guidelines for clinical trials^38^.

Our study represents MPE research that we examined the relationship between the *FAS* susceptibility alleles and its aberrant transcriptional activities by ChIP assays and finally revealed the function of these alleles and gained insight into whether susceptibility alleles were truly causal. Recently, a new direction of MPE where researchers investigate the interactive effects of tumorous molecular features and the exposures of interest on tumor behavior (prognosis or clinical outcome) has emerged. These studies will help us to attribute the effects of exposure of interest to a specific molecular subtype of cancer.

In conclusion, the *FAS* rs2234767 and rs1800682 polymorphisms were in high LD with each other, and they jointly contributed to an increased risk and progression of CRC. The interaction between them altered the the ability of SP1 and STAT1 transcription factors to bind the endogenous *FAS* promoter region as assessed by ChIP assays. These findings suggest that the functional promoter polymorphisms of *FAS* may jointly contribute to the etiology of CRC.

## Additional Information

**How to cite this article**: Wang, S. *et al. FAS* rs2234767 and rs1800682 polymorphisms jointly contributed to risk of colorectal cancer by affecting SP1/STAT1 complex recruitment to chromatin. *Sci. Rep.* **6**, 19229; doi: 10.1038/srep19229 (2016).

## Supplementary Material

Supplementary Information

## Figures and Tables

**Table 1:** Association between *FAS* rs2234767, rs1800682 and *FASLG* rs763110 genotypes and risk of CRC.

SNPs	Genotype	Cases (n = 878)		Controls (n = 884)		*P*^a^	*P*^b^	Adjusted OR (95% CI)^c^
		n	%	n	%			
*FASLG*	CC	462	53	470	53	0.71	1.0	1.00 (Ref)
rs763110	CT	354	40	344	39			1.06 (0.87–1.28)
	TT	62	7	70	8			0.90 (0.62–1.30)
	*P*_trend_							0.92
	T allele	478	27	484	27	0.92	1.0	
*FAS*	GG	305	37	385	44	**0.0051**	**0.015**	1.00 (Ref)
rs2234767	GA	407	49	372	43			**1.39 (1.13–1.71)**
	AA	124	15	114	13			**1.37 (1.02–1.84)**
	*P*_trend_							0.0051
	A allele	655	39	600	34	**0.0042**	**0.013**	
	GG	305	36	385	44			1.00 (Ref)
	GA/AA	531	64	486	56			**1.38 (1.14–1.68)**
*FAS*	AA	301	34	348	40	0.060	0.18	1.00 (Ref)
rs1800682	AG	435	50	392	44			1.30 (1.06–1.60)
	GG	142	16	144	16			1.16 (0.88–1.53)
	*P*_trend_							0.14
	G allele	719	41	680	38	0.13	0.39	
	AA	301	34	348	39			1.00 (Ref)
	AG/GG	577	66	536	61			**1.26 (1.04–1.53)**

Bold indicated statistically significant.

^a^χ^2^ test for either genotype distributions or allele frequencies between the cases and controls.

^b^Adjusted for multiple comparisons by Bonferroni correction.

^c^Adjusted for age, sex, smoking and drinking status in logistic regression model.

**Table 2:** Combined genotype frequencies of the *FAS* polymorphisms among the cases and controls and their association with risk of CRC.

Combined genotypes		Cases (n = 836)	Controls (n = 871)	*P*^a^	Adjusted OR (95% CI)^b^
		n (%)	n (%)		
*FAS* rs2234767	*FAS* rs1800682			**<0.001**	
GG	AA	287 (34)	347 (40)		1.00 (Reference)
GG	AG/GG	18 (2)	38 (3.9)		0.60 (0.33–1.07)
GA/AA	AA	13 (2)	1 (0.1)		**15.49 (2.01–119.25)**
GA/AA	AG/GG	518 (62)	485 (56)		**1.30 (1.06–1.59)**
Trend test				**0.0051**	

Bold indicated statistically significant.

^a^χ^2^ test for the combined genotype distributions between the cases and controls.

^b^Adjusted for age, sex, smoking and drinking status in logistic regression model.

**Table 3:** Stratified analysis of the *FAS* combined genotypes associated with CRC risk by demographic variables.

Variables	Case/control (n)	Combined genotypes (case/control)				*P*^a^	Adjusted OR (95% CI)^b^
		With rs2234767 GG/rs1800682 AA		Without rs2234767 GG/rs1800682 AA			
		n	%	n	%		
Total	836/871	287/347	34/40	549/524	66/60	**0.019**	**1.28 (1.05–1.56)**
Age (years)							
≤60	436/401	156/156	36/39	280/245	64/61	0.35	1.09 (0.82–1.45)
>60	400/470	131/191	33/41	269/279	67/59	**0.016**	**1.50 (1.13–1.99)**
Sex							
Male	510/507	198/207	39/41	312/300	61/59	0.51	1.08 (0.84–1.39)
Female	326/364	89/140	27/38	237/224	73/62	**0.0019**	**1.77 (1.26–2.47)**
Smoking status							
Never	558/602	185/244	33/40	373/358	67/60	**0.0093**	**1.39 (1.09–1.77)**
Ever	278/269	102/103	37/38	176/166	63/62	0.70	1.08 (0.76–1.53)
Drinking status							
Never	610/655	207/264	34/40	403/391	66/60	**0.019**	**1.33 (1.05–1.67)**
Ever	226/216	80/83	35/38	146/133	65/62	0.51	1.16 (0.78–1.72)
Family history of cancer							
No	642/786	222/323	35/41	420/463	65/59	**0.012**	**1.34 (1.08–1.67)**
Yes	194/85	65/24	34/28	129/61	66/72	0.39	0.78 (0.44–1.37)

Bold indicated statistically significant.

^a^χ^2^ test for the combined genotype distributions between the cases and controls.

^b^Adjusted for age, sex, smoking and drinking status in logistic regression model.

**Table 4:** Association between the *FAS* combined genotypes and progression of CRC.

Variables	Combined genotypes				*P*^a^	Adjusted OR (95% CI)^b^
	With rs2234767 GG/rs1800682 AA		Without rs2234767 GG/rs1800682 AA			
	n	%	n	%		
Controls (n = 871)	347	40	524	60		1.00 (reference)
Cases (n = 836)						
Tumor grade						
Low	24	40	36	60	0.98	0.99 (0.58–1.70)
Intermediate	219	34	427	66	**0.018**	**1.30 (1.05–1.61)**
High	44	34	86	66	0.19	1.30 (0.88–1.91)
Dukes stage						
A + B	150	35	275	65	0.11	1.22 (0.96–1.55)
C + D	137	33	274	67	**0.025**	**1.33 (1.04–1.71)**

Bold indicated statistically significant.

^a^χ^2^ test for the combined genotype distributions between the cases and controls.

^b^Adjusted for age, sex, smoking and drinking status in logistic regression model.
