# Supplementary material for: FAS rs2234767 and rs1800682 polymorphisms jointly contributed to risk of colorectal cancer by affecting SP1/STAT1 complex recruitment to chromatin
Source: Sci Rep. 2016 Jan 13;6:19229. doi: 10.1038/srep19229 (PMC4725756; doi:10.1038/srep19229)
Supplement: Supplementary Information [file srep19229-s1.pdf]

FAS rs2234767 and rs1800682 polymorphisms jointly contributed to risk of colorectal cancer by affecting SP1/STAT1 complex recruitment to chromatin

Shizhi Wang, Shenshen Wu, Qingtao Meng, Xiaobo Li, Rui Chen, Meilin Wang

Figure legends.

Fig. S1. _FAS_ rs2234767A and rs1800682G alleles affect coupled SP1 and STAT1 recruitment to chromatin. (A) Chromatin immunoprecipitation (ChIP) of the *FAS* promoter with three different genotypes (rs2234767GG/rs1800682AA, rs2234767GA/rs1800682AG and rs2234767AA/rs1800682GG) using antibody for SP1 and STAT1(single pool generated from triplicate biological samples/manipulation; triplicate measurements/pool; mean ± SE). (B) Sequential ChIP of the *FAS* promoter containing SP1 motif immunoprecipitated first using antibody for SP1 followed by antibody for STAT1 (upper) or first using antibody for STAT1 followed by antibody for SP1 (lower). (C) Sequential ChIP of the *FAS* promoter containing STAT1 motif immunoprecipitated first using antibody for SP1 followed by antibody for STAT1 (upper) or first using antibody for STAT1 followed by antibody for SP1 (lower). For all combined genotypes in the figure, left genotype arises from the rs2234767 and right from the rs1800682 polymorphism.

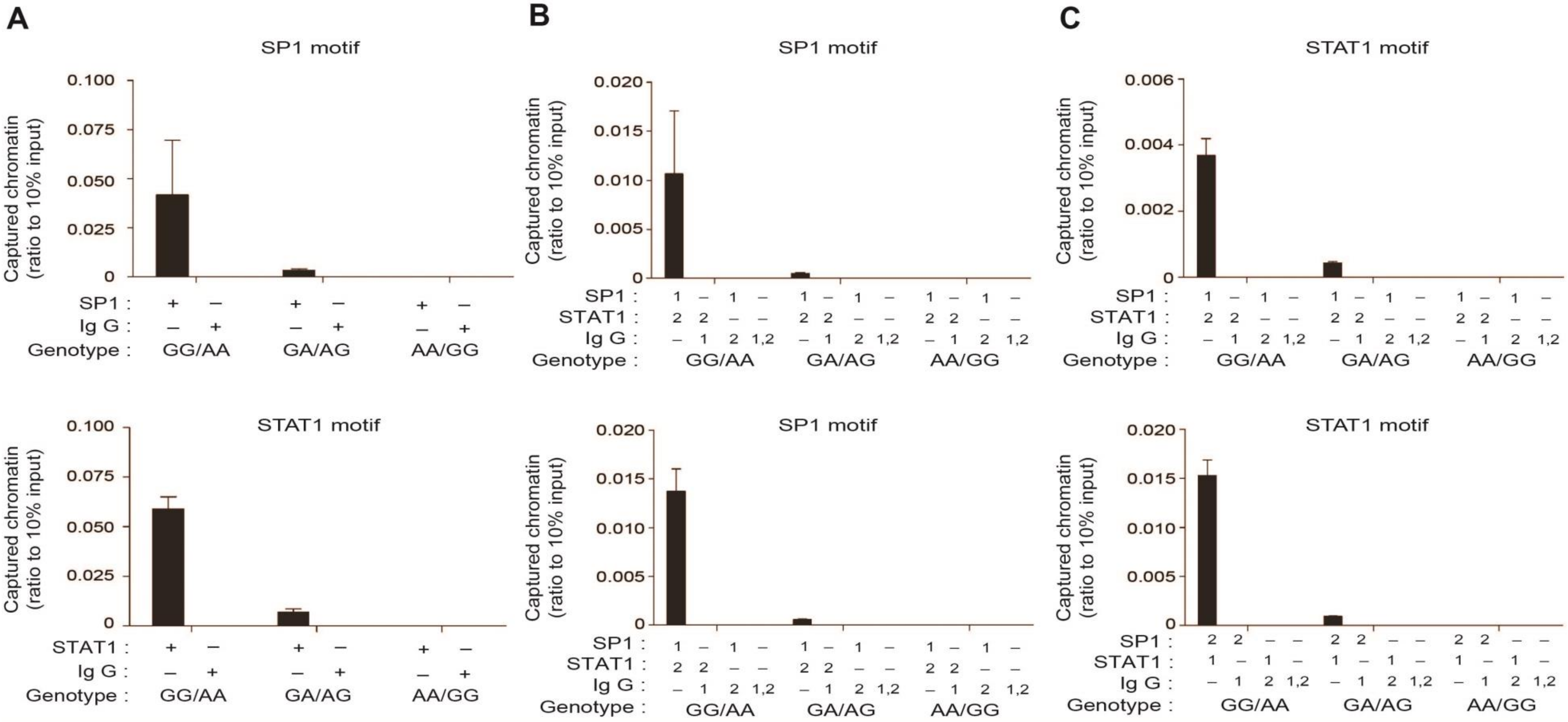
A
SP1 motif
Captured chromatin (ratio to 10% input)
0.100
0.075
0.050
0.025
0
SP1 : + – + – + –
Ig G : – + – + – +
Genotype : GG/AA GA/AG AA/GG
STAT1 motif
STAT1 : + – + – + –
Ig G : – + – + – +
Genotype : GG/AA GA/AG AA/GG
B
SP1 motif
0.020
0.015
0.010
0.005
SP1 : 1 – 1 – 1 – 1 – 1 – 1 –
STAT1 : 2 2 – – 2 2 – – 2 2 – –
Ig G : – 1 2 1,2 – 1 2 1,2 – 1 2 1,2
Genotype : GG/AA GA/AG AA/GG
C
STAT1 motif
0.006
0.004
0.002
SP1 : 1 – 1 – 1 – 1 – 1 – 1 –
STAT1 : 2 2 – – 2 2 – – 2 2 – –
Ig G : – 1 2 1,2 – 1 2 1,2 – 1 2 1,2
Genotype : GG/AA GA/AG AA/GG
SP1 : 2 2 – – 2 2 – – 2 2 – –
STAT1 : 1 – 1 – 1 – 1 – 1 – 1 –
Ig G : – 1 2 1,2 – 1 2 1,2 – 1 2 1,2
Genotype : GG/AA GA/AG AA/GG
